# Connecting aging biology and inflammation in the omics era

**DOI:** 10.1172/JCI158448

**Published:** 2022-07-15

**Authors:** Keenan A. Walker, Nathan Basisty, David M. Wilson, Luigi Ferrucci

**Affiliations:** 1 Intramural Research Program of the National Institute on Aging, NIH, Baltimore, Maryland, USA.; 2 Biomedical Research Institute, Faculty of Medicine and Life Sciences, Hasselt University, Diepenbeek, Belgium.

## Abstract

Aging is characterized by the accumulation of damage to macromolecules and cell architecture that triggers a proinflammatory state in blood and solid tissues, termed inflammaging. Inflammaging has been implicated in the pathogenesis of many age-associated chronic diseases as well as loss of physical and cognitive function. The search for mechanisms that underlie inflammaging focused initially on the hallmarks of aging, but it is rapidly expanding in multiple directions. Here, we discuss the threads connecting cellular senescence and mitochondrial dysfunction to impaired mitophagy and DNA damage, which may act as a hub for inflammaging. We explore the emerging multi-omics efforts that aspire to define the complexity of inflammaging — and identify molecular signatures and novel targets for interventions aimed at counteracting excessive inflammation and its deleterious consequences while preserving the physiological immune response. Finally, we review the emerging evidence that inflammation is involved in brain aging and neurodegenerative diseases. Our goal is to broaden the research agenda for inflammaging with an eye on new therapeutic opportunities.

Aging has been conceptualized as a continuous duel between damage accumulation — due to a combination of environmental and endogenous processes — and resilience mechanisms that cope with such stressors and resolve damage (1). With aging, resilience mechanisms become less effective at repairing or removing damage and preventing its deleterious effects on health (2). Persistent molecular and cellular damage due to exhausted resilience is ultimately expressed as phenotypes of aging, including inflammaging, susceptibility to chronic diseases, physical and cognitive impairments, and, ultimately, frailty and death.

Atop the hierarchy of resilience is the immune system, the aggregate of cells, mediators, and signaling pathways that continuously patrol for pathogens or structural perturbations revealed as “unusual” molecular motifs. The immune system reacts to a variety of threats, such as symbiotic commensal and pathogenic microorganisms, pathogen-associated molecular patterns (PAMPs), and damage-associated molecular patterns (DAMPs) from endogenous and exogenous sources, and orchestrates defense responses aimed at eliminating the specific threat while minimizing damage to the host. While inflammation is important for tissue repair and regeneration, when abnormally intense or persistent, it can drive degeneration and chronic diseases.

The immune system undergoes numerous and profound changes with aging, which are extensively reviewed elsewhere (3–5). Hallmarks of immune aging are (a) a state of proinflammatory activation characterized by high circulating levels of proinflammatory cytokines — such as IL-6 and TNF-α — and localized tissue inflammation, and (b) an aberrant response to antigens and pathogens that could either be blunted, such as in flu vaccination, or excessive, such as in response to SARS-CoV-2 (6).

Considerable research in both animal models and humans has examined the causes and consequences of inflammaging (4). Although increased levels of inflammatory mediators (mostly IL-1, IL-6, TNF-α, and its receptors) are detected in all elderly individuals, higher levels of these biomarkers are associated with increased risk for many chronic conditions, including dementia, disability, and physical frailty. Inflammation’s causal role in cardiovascular disease was established by the CANTOS trial (Canakinumab Anti-Inflammatory Thrombosis Outcomes Study), which demonstrated that IL-1β inhibition reduced the risk of cardiovascular events versus the placebo, particularly in participants whose IL-6 levels were initially elevated (7).

Mechanisms identified as hallmarks of aging biology and immune cell dysfunction have all been hypothesized as causes of inflammation (8). Aging researchers now recognize that measuring a few cytokines in circulation fails to capture the complexity and potential ramifications of inflammaging. Immune cells in tissues, particularly lymphocytes and resident macrophages, show tissue-specific age-related changes likely connected to specific pathological processes (9). By measuring hundreds or thousands of molecules in a few drops of blood, scientists are attempting to identify (a) signatures of accelerated aging that are both informative of the complexity and diversity of the response and predictive of health outcomes and (b) key molecules and molecular mechanisms that can be targeted for intervention (10).

Given the extreme complexity of inflammaging, we focus herein on a few topics that have attracted considerable attention and controversy in the field. First, we discuss cellular senescence as a source of local and systemic inflammation. We highlight evidence that mitochondrial dysfunction is a nexus that binds impaired mitophagy with DNA damage and cellular senescence to ultimately foster a chronic inflammatory state. We then summarize efforts to identify circulating signatures of inflammation through “omics.” Finally, we review emerging data indicating that inflammation is involved in brain aging and dementia. Our intent is to discuss the causes and consequences of inflammaging and to enrich the research agenda toward the development of new therapeutic strategies.

## Senescent cells in the inflammation-aging axis

Senescent cells and the senescence-associated secretory phenotype (SASP), now widely acknowledged as drivers of aging and age-related diseases, have emerged as key players in inflammaging. Cellular senescence can be initiated by multiple intrinsic and extrinsic nonlethal stresses, including genotoxic, oncogenic, mitochondrial, oxidative, inflammatory, paracrine signaling, and others (11). Hallmarks of cellular senescence include growth and replication arrest, resistance to apoptosis, chromatin remodeling, metabolic reprogramming, and morphological changes, among others (11–13). While highly specific criteria for defining cellular senescence are still debated, it is generally believed that all senescent cells secrete a set of diverse bioactive molecules, both soluble and within extracellular vesicles, known as the SASP. The SASP includes proteases, growth factors, and, notably, a collection of cytokines, chemokines, and other inflammatory mediators (14, 15). Via the SASP, senescent cells contribute to multiple chronic health conditions associated with inflammaging, e.g., atherosclerosis (16), osteoarthritis (17), cancer (18), myeloid skewing in the bone marrow (19), pulmonary fibrosis (20), Alzheimer’s disease and Parkinson’s disease (21–23), and insulin resistance (24). We detail next the contribution of senescent cells to inflammaging, how the aging immune system may contribute to senescent cell accumulation in peripheral tissues, and how assessing the burden of senescent cells in different tissues through “omics” may aid in the development of strategies to alleviate diseases associated with inflammaging.

### Emerging role of senescence in the immune system.

Cells of the immune system can undergo cellular senescence or enter a senescent-like state (25–29). Yousefzadeh and colleagues recently suggested a causative link between a senescent immune system and nonlymphoid tissue aging (26). Deletion of ERCC1, a crucial DNA repair protein, in mouse hematopoietic cells leads to accelerated senescence of the immune system. Accordingly, mice lacking ERCC1 in hematopoietic cells, as well as aged mice, express elevated markers of senescence (p16, p21) in many immune cells with concomitant impairment of immune function. Strikingly, transfer of splenocytes from aged wild-type mice or mice lacking ERCC1 into young animals increased the senescence burden in nonlymphoid organs, suggesting that a senescent immune system contributes to aging phenotypic features in immune cells and in solid, nonlymphoid tissues. Thus, senescent immune cells may be central upstream targets for treating inflammaging-associated phenotypes and reducing systemic senescent cell burden.

Knowing whether senescent immune cells induce cellular senescence in peripheral tissues is central to designing strategies of prevention and treatment. T cell populations exhibiting multiple senescence markers and lacking proliferative capacity, in addition to B cells (30) and monocytes (31) that carry senescent features, have been described (32) and implicated in immunopathology. Accessible in circulation, senescent immune cells may prove easier to therapeutically eliminate than tissue-resident ones. Furthermore, owing to their proximity to blood, factors secreted into plasma by circulating senescent cells are more likely to be detectable and measurable in biomarker studies and be therapeutically targeted. Unfortunately, non-senescent immune cells can express and secrete, usually to a lesser degree, some of the same markers used to identify senescent cells, and research is needed to identify markers with the best discriminative value. For example, certain “senescent-like” macrophages showing high expression of p16^INK4a^ and senescence-associated β-galactosidase (SA-β-gal) activity accumulate during aging, yet immunomodulatory agents can reverse their expression of these senescence markers (27, 28). Isolated p16^INK4a^-expressing cells from in vivo, however, express a range of senescence and SASP markers (29). These findings suggest that the beneficial effects of eliminating p16-positive cells are due solely to elimination of senescent cells, non-senescent macrophages, or some combination thereof.

### Circulating SASP mediates senescent cell–immune system interactions.

The production of SASP inflammatory factors is primarily driven by NF-κB, a rapid-acting transcription factor that orchestrates the response to a range of cellular stressors (33). A p21/Rb pathway also produces a p21-activated secretory phenotype (PASP) that directs a CXCL14-mediated mechanism of senescent cell immunosurveillance (34). Many of the SASP components can activate and recruit cells of the immune system and drive inflammation, including the prototypical markers of chronic sterile inflammaging, i.e., IL-6, IL-1, and TNF-α (14, 35). Since a variety of circulating SASP factors increase with age, likely leaked from tissue-resident senescent cells, a subset of SASP factors have been proposed as biomarker candidates of aging and age-related diseases: growth differentiation factor 15 (GDF15), stanniocalcin 1 (STC1), matrix metalloproteinase 1 (MMP-1), inhibin subunit βA (also known as activin A, INHBA), TNF receptor superfamily member 1A (TNFRSF1A), and serpin family E member 1 (SERPINE1) (15, 35). Epidemiological studies indeed indicate that proteomic SASP panels predict chronological age, biological age, mortality, morbidity, and adverse health outcomes (35–37). High senescent cell burden also drives hematological phenotypes in mice, including blood clotting, which can be reversed upon elimination of senescent cells (38). Additionally, senescence occurs at sites of atherosclerotic plaques, where it participates in multiple stages of atherogenesis (16, 39) and, through a paracrine SASP mechanism, accelerates the atherosclerotic process. These observations suggest that the SASP is secreted into the blood at sufficient levels to contribute to systemic inflammaging and for detection. Consistent with this notion, the administration of dasatinib and quercetin (D+Q), a well-studied senolytic cocktail, in subjects with diabetic kidney disease reduced senescent cell numbers and circulating levels of SASP factors, such as IL-1α and IL-6 (20, 40–45). These initial findings are promising, but further studies are necessary (a) to confirm that the SASP in human biofluids correlates with senescent cell burden in tissues, (b) to identify the subset of SASP factors that are most representative of senescence and disease burden in various tissues, and (c) to identify features of the secretory phenotype that are potentially contributed by sources other than senescent cells.

**Figure 1:**
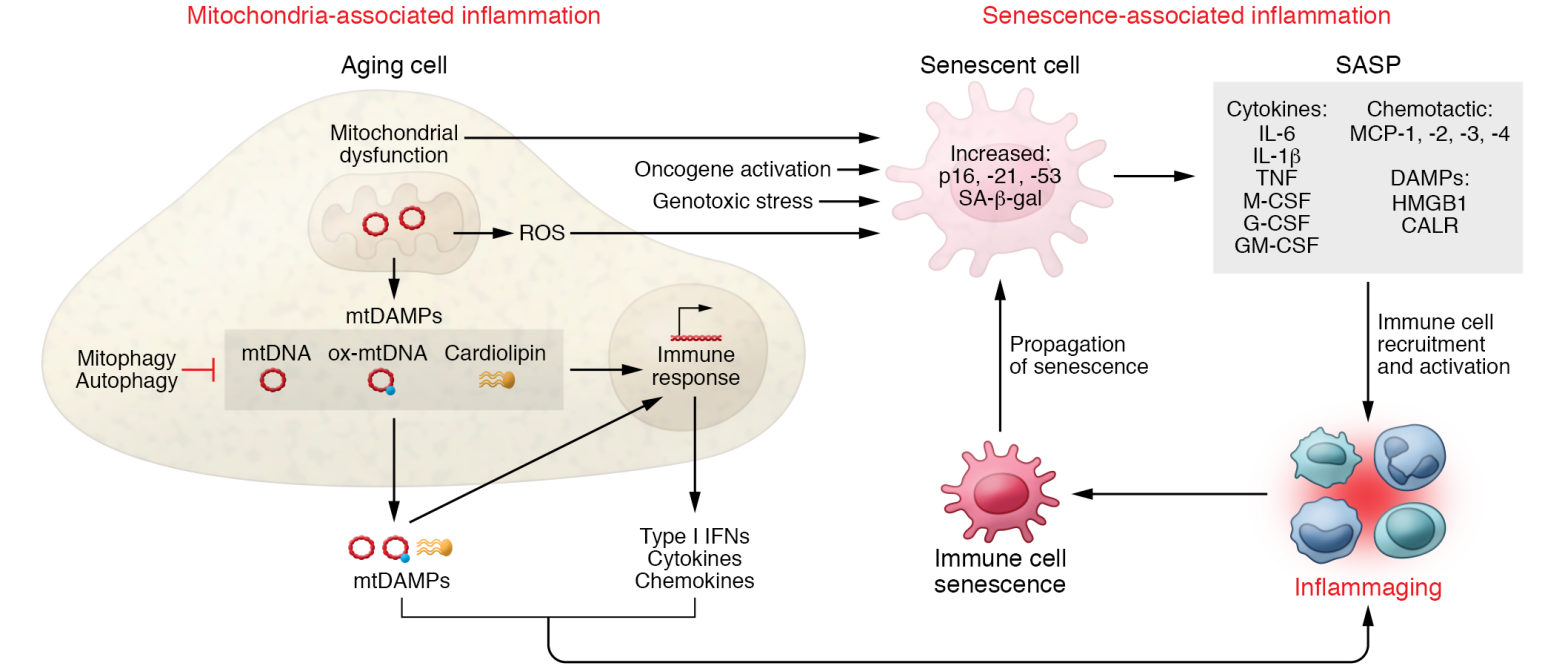
Interplay between cellular senescence, mitochondrial dysfunction, and the aging immune system. During aging, increasing dysfunctional mitochondria contribute to inflammation through several pathways and interact with other hallmarks of aging such as cellular senescence and autophagy. Mitochondrial-derived damage-associated molecular patterns (mtDAMPs) accumulate in the cytosol, including mitochondrial DNA (mtDNA), oxidized mtDNA (ox-mtDNA), and cardiolipin. Accumulation of mtDAMPs leads to downstream production of inflammatory cytokines, type I interferons (IFNs), and chemokines via intracellular pattern recognition receptors to activate an immune response. The collective secretion of mtDAMPs, IFNs, cytokines, and chemokines into the extracellular environment contributes to inflammaging. With age, an increasing mitochondrial dysfunction and production of ROS lead to increased levels of senescent cells, which accumulate naturally with age and in response to multiple stimuli. The accumulation of senescent cells and the SASP increases the release of bioactive molecules, including cytokines, recruitment factors, and DAMPs, into circulation, thereby driving inflammaging. In turn, the accumulation of senescent lymphocytes in the aging immune system further propagates cellular senescence in peripheral tissues.

The SASP directly affects the innate and adaptive immune systems (Figure 1) by promoting recruitment, differentiation, and/or activation of natural killer cells, macrophages, monocytes, neutrophils, and T lymphocytes (46). For instance, the SASP factors granulocyte colony-stimulating factor (G-CSF), macrophage colony-stimulating factor (M-CSF), and granulocyte-macrophage colony-stimulating factor (GM-CSF) can stimulate the production and activity of monocytes and their differentiation into macrophages. The SASP also connects aging to immunometabolism, promoting the proliferation of the CD38^+^ population of macrophages that contribute to the age-associated decline of NAD^+^ (47). Moreover, SASP monocyte chemoattractant proteins (MCPs) and other cytokines/chemokines stimulate the activation and tissue infiltration of monocytes and macrophages. For example, injection of alginate-encapsulated senescent cells into mice causes the recruitment of M2-like resident peritoneal macrophages surrounding the capsules (27). Additionally, senescent fibroblasts in aged skin recruit proinflammatory monocytes by secreting MCP-1 (48). SASP factors may also blunt T cell responses in aged individuals by promoting the secretion of T cell–suppressing prostaglandin E_2_ (PGE_2_), implicating the SASP in the age-related decline of adaptive immunity (48).

Senescent cells, including senescent fibroblasts, secrete DAMPs, which constitute endogenous molecules released from cells in response to internal and external stressors (15, 49). DAMPs that may trigger chronic sterile inflammation during aging and age-related pathologies include high-mobility group box 1 (HMGB1) and calreticulin (CALR) (50).

### Translation to humans.

Massive efforts are under way to develop senotherapies that selectively eliminate senescent cells (senolytics) and/or modulate the secretory phenotype (senomorphics) as a strategy to mitigate inflammaging and its consequences. Several senolytics reduce or improve inflammatory or age-related phenotypes in preclinical models, specifically atherosclerosis (D+Q, navitoclax) (16, 41), osteoarthritis (navitoclax) (17), idiopathic pulmonary fibrosis (D+Q), and insulin resistance (23), among others, as well as rejuvenating hematopoietic stem cells in aged mice (navitoclax) (19). Cellular senescence is a driver of both physiologically beneficial phenotypes and age-related pathologies owing to the heterogeneous phenotypes of senescent cells that inhabit an organism or tissue, i.e., “senotype,” which is dependent on biological context and environmental factors. Achieving senotype specificity, or tailoring senolytics to harmful subsets of senescent cells while sparing potentially beneficial ones, presents a challenge to be addressed by future generations of senolytics (51, 52).

An aforementioned complexity of senotherapies is that cellular senescence has beneficial roles in wound healing, tumor suppression, and embryonic development, while also driving inflammaging (53). Removal of some senescent cell types can adversely affect wound healing and fibrosis of liver and perivascular tissue (54, 55). Attempts to distinguish between beneficial and pathological cell senescence at the proteome (15), transcriptome (56, 57), and functional levels have not been successful to date. Studies that capture the complex and dynamic nature of senescent cells by profiling across heterogeneous conditions are needed to identify precise senotype-specific biomarkers and to track the efficacy of interventions. In recent years, unbiased and comprehensive proteomic studies have uncovered an expanded list of factors that may help develop a signature of “senescence burden” in human plasma encompassing inflammatory factors (CXCLs, HMGB1, PTX3, etc.), growth factors (GDF15, IGFBPs), extracellular matrix–associated (ECM-associated) or ECM-modifying components (MMPs, TIMPs, CD44), and tailored sets of robustly expressed age-associated proteins (15). Looking forward, senolytic trials that precisely assess senescence burden with multi-omic and senotype-specific biomarker signatures are needed to identify the most effective senolytic drugs to combat inflammaging-associated pathologies.

## Age-related mitochondrial dysfunction and inflammaging

Mitochondria provide the energy essential for all cellular activities, including the fueling of resilience mechanisms that counteract macromolecule damage accumulation with aging (1). Balancing between a cell’s energy/metabolic requirements and energy availability, mitochondria trigger adaptive responses and stress signals, including ROS production and activation of innate immunity. Mitochondrial function declines with aging in model organisms and humans (58). Impaired mitochondrial function, which involves excessive ROS and reduced ATP production, has been associated with visceral obesity, insulin resistance, higher circulating levels of proinflammatory markers, and loss of mobility, phenotypes that are characteristic of aging (59–64). Physical activity improves mitochondrial function in multiple tissues and reverses many features of aging, indicating the importance of efficient mitochondrial activity in preserving health and longevity (65).

Inflammation driven by impaired mitochondrial function occurs in the context of dysfunction in other aging hallmarks. Genomic instability, defective autophagy/mitophagy, and cellular senescence, three major hallmarks of aging, are intertwined with mitochondria-related inflammatory responses (Figure 1) (66). DNA damage accumulates with age and contributes to age-related phenotypic changes (67). DNA of mammal cells experiences over 10^5^ modifications per day, many of which are ROS-related single-strand breaks that are detected by the DNA-break sensor poly(ADP-ribose) polymerase 1 (PARP1), and ultimately resolved by defined repair mechanisms (68). Persistently activated PARP1, however, consumes large amounts of NAD^+^, an essential coenzyme of sirtuins (SIRTs) (69). Depletion of NAD^+^ causes loss of efficient sirtuin activity, resulting in impaired functionality of mitochondrial SIRT3, leading to dysregulation in mitochondrial antioxidant systems, mitochondrial DNA (mtDNA) repair, and mitochondrial quality control and biogenesis (SIRT1) pathways, and may also promote senescence (70). Reduced NAD^+^ levels also impair SIRT2 activity, which normally deacetylates and inactivates the NLR family pyrin domain containing 3 (NLRP3) inflammasome, thereby counteracting inflammation (71). Indeed, NAD^+^ supplementation mitigates premature aging phenotypes associated with defects in DNA repair by restoring mitochondrial function and mitophagy (72, 73). Conversely, abnormal autophagy and mitophagy, often observed in chronic disease and aging, fail to remove dysfunctional mitochondria that release DAMPs and ROS, activating NLRP3 and mitochondria-related inflammation (74).

**Figure 2:**
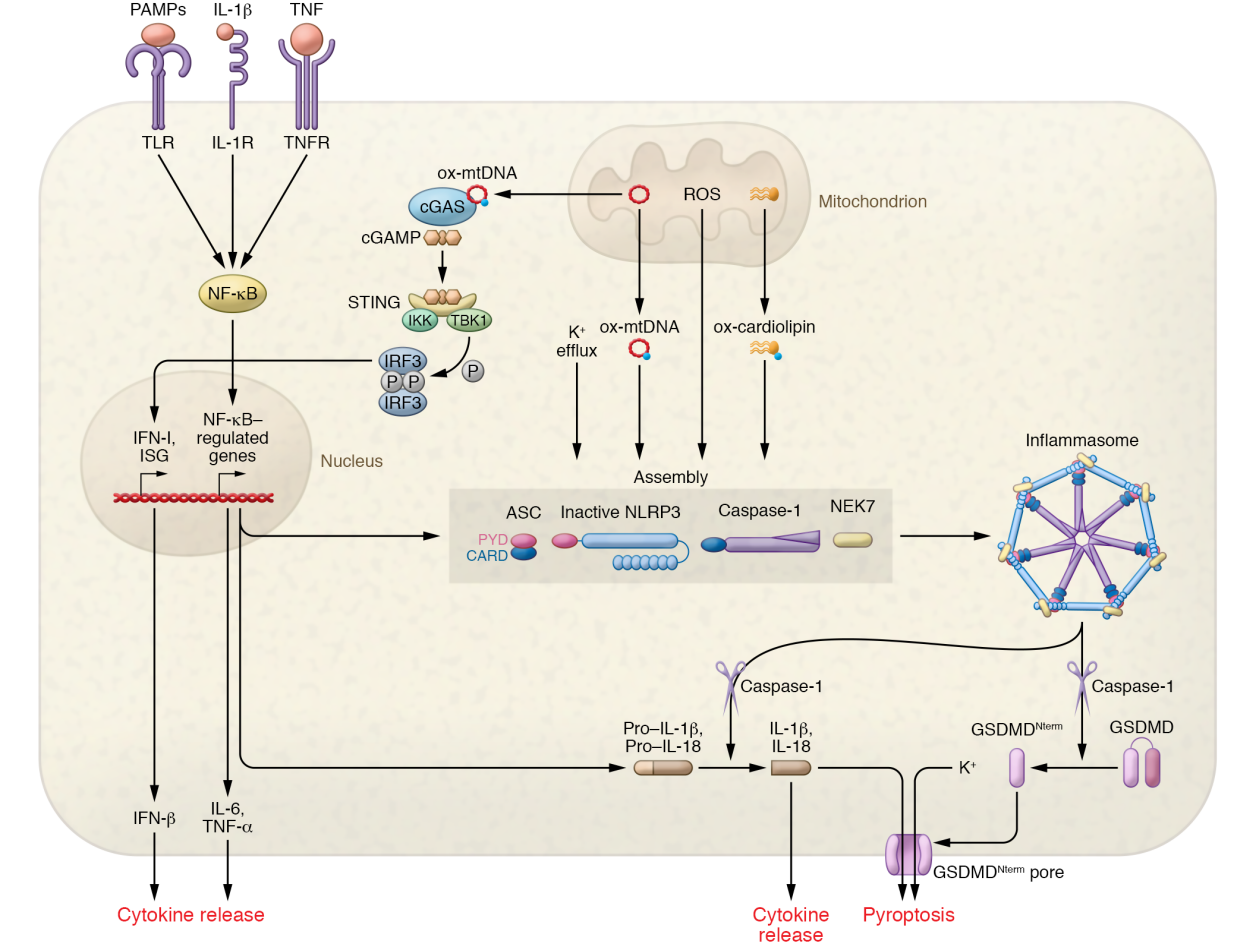
Mitochondria are hubs of inflammation. Mitochondria are at the center of three inflammatory mechanisms: the NLRP3 inflammasome, cGAS/STING pathway, and NF-κB pathway. NLRP3 inflammasome activation is considered a two-step process: First, priming requires activation of NF-κB by PAMPs or DAMPs binding to pattern recognition receptors including TLRs or by cytokines, such as TNF-α or IL-1β, via their receptors. NF-κB stimulates the transcription and posttranslational stabilization of inactive forms of NLRP3, precursors of cytokines pro–IL-1β and pro–IL-18, and other NF-κB–related cytokines such as IL-6. Second, during activation, a multitude of DAMPs, such as ATP, cholesterol crystals, and urate crystals, promote inflammasome assembly, involving NIMA-related kinase 7 (NEK7), a sensor of K^+^ efflux (180). The activation likely requires K^+^ efflux, ROS production, and relocalization of cardiolipin to the outer mitochondrial membrane. It has been proposed that all stimuli that trigger the NRLP3 inflammasome converge on mitochondria, possibly via release of newly synthesized oxidized mtDNA, but K^+^ efflux may also be critical (181, 182). NLRP3 activation causes activation of caspase-1 and cleavage of pro–IL-1β and pro–IL-18 to their activated forms. Caspase-1 also cleaves gasdermin D (GSDMD), which oligomerizes to form pores in the cell membrane, which may lead to extracellular release of IL-1 and IL-18, K^+^ efflux, and massive swelling with membrane rupture (pyroptosis). The DNA sensor cGAS binds to mtDNA released into the cytosol and catalyzes production of the secondary messenger cyclic GMP-AMP (cGAMP) from ATP and GTP. cAGMP binds to the ER membrane adaptor STING, which is displaced to the perinuclear endosome and activates TANK binding kinase 1 (TBK1), which, in turn, phosphorylates interferon regulatory factor 3 (IRF3) that enters the nucleus and enhances transcription of type I IFN and IFN-stimulated genes (ISGs), while also activating NF-κB. In cell culture, cGAS overexpression induces IFN-β, whereas cGAS knockdown erases IFN-β induction by DNA transfection (183). cGAS/STING activation also enhances autophagy (184) and induces cellular senescence (185). NF-κB is activated by diverse stress signals and pathways related to defense and survival (89, 186). Multiple stress signals activate the NF-κB dimer (composed of p65/p50 subunits) that translocates to the nucleus, where it binds to consensus sequences in regulatory regions of target genes (187). NF-κB activation drives multiple pleiotropic effects, including enhancing the transcription of proinflammatory mediators, such as TNF-α and IL-6, that regulate both innate and adaptive immunity (95). CARD, caspase recruitment domain; GSDMD^Nterm^, GSDMD amino-terminal cell death domain; LRR, leucine-rich repeat; PYD, pyrin domain.

In physiological conditions, ROS operate as signaling molecules for diverse cellular functions and are rapidly quenched by antioxidants, such as thioredoxin and glutathione (75). However, impairments in oxidative phosphorylation increase the release of ROS, causing oxidative damage to macromolecules and ultimately cell dysfunction and death (76). Impaired mitochondria release DAMPs, composed of mostly oxidatively modified mtDNA and cardiolipin, into the cytosol through apoptosis-activated BAK/BAX macropore assemblies (77). mtDNA harbors conserved unmethylated CpG motifs characteristic of bacterial DNA, and when it enters the cytosol, it is sensed as “non-self” by the innate immune system via pattern recognition receptors, such as TLR9 and NLRs (78). Cardiolipin — a phospholipid that is normally segregated to the inner mitochondrial membrane and is essential to the maintenance of mitochondrial architecture and function — when herniated outside a damaged mitochondrial membrane, stimulates mitophagy, a process that eliminates dysfunctional mitochondria and promotes the formation of new mitochondria (79). In most severe cases, cardiolipin stimulates the release of cytochrome *c* and precipitates apoptosis (80). When ROS-damaged mtDNA and cardiolipin are released into the cytosol, various inflammatory pathways are activated. Responses that have been best studied involve the NLRP3 inflammasome, the DNA-sensing cGAS/STING pathway, and NF-κB, which are described in further detail in Figure 2.

Studies have demonstrated important roles of the NLRP3 inflammasome in aging and age-related diseases, such as atherosclerosis metabolic syndrome, type 2 diabetes, and Alzheimer’s disease (81–83). Autophagy can clear mitochondria-related DAMPs, as well as pro–IL-1β and ubiquitinated inflammasome complexes, thereby reducing the proinflammatory environment (84). Indeed, autophagy inhibition increases free mtDNA in the cytosol and the production of IL-1β (85). Ablation of the NLRP3 inflammasome protects mice from age-related increases in innate immune activation, alterations in the CNS transcriptome, and astrogliosis, while improving glycemic control and motor performance (86). Loss-of-function mutations in the *STING* gene are protective from aging-related diseases, especially among smokers affected by chronic pulmonary diseases, who typically have a proinflammatory state (87). Conversely, rare *STING* gain-of-function mutations are associated with massive inflammation especially in lungs (88). NF-κB complexes have been found inside mitochondria, juxtaposed to the inner membrane, and there is evidence that the NF-κB pathway is activated during mitochondrial dysfunction and oxidative stress, although the mechanism of activation is not defined (89). The canonical NF-κB pathway is triggered by both endogenous factors and environmental factors, including diet and air pollution, mostly via stimulation of proinflammatory receptors, such as the TNF receptor superfamily and the IL-1 receptor (90). NF-κB activation has been observed in numerous diseases associated with aging (91), including sarcopenia (92), osteoporosis (93), and neurodegenerative diseases (94).

## Application of multi-omics approaches to study inflammaging

Progress has been made in understanding the complex relationship between aging and acute and chronic inflammation mostly due to the expansion of tools available to measure and quantify immune function and biomarkers. In vivo characterization of inflammation in humans has been particularly useful in revealing the nuances of biological responses to different stimuli (95). The emerging picture is that inflammation is orchestrated by a large number of cell types and mediators and takes on a wide range of forms to best address the specific challenge (96). Inflammation covers a wide spectrum of biological manifestations that are dictated by the inflammatory stimulus, the stage in the immune response, and a variety of person-specific host factors. The challenge is using biomarkers to distinguish between different nuances of inflammation and to determine how responses change with aging so as to pinpoint more precise therapeutic avenues that effectively reduce the burden of age-related disease.

Efforts to characterize inflammation in humans have relied on one or a few inflammatory proteins, with IL-6, TNF-α, and CRP being the most widely used (5). The blood and tissue level of these proteins increases with age (97) and, to varying degrees, has been associated with adverse age-related outcomes, including physical frailty (98), cardiovascular disease (7, 99), dementia (100), and mortality (101). It is now understood that these proteins represent only a component of inflammaging (102). Because of the complexity and pleiotropic nature of inflammatory signaling cascades, and the dynamic expression of inflammatory proteins following immune stressors, measuring only a single or a small number of inflammatory proteins increases the likelihood that contradictory results will be obtained from otherwise comparable studies. Multi-omics and multiplexing technology have provided investigators with necessary tools to characterize inflammation with a higher degree of resolution.

Multiplex platforms that assess an array of inflammatory indicators are increasingly used to characterize the multidimensional nature of inflammation. By pairing measures from multiple platforms, including transcripts, epigenetic modulators, proteins, and metabolites, with supervised or unsupervised dimension reduction approaches, investigators are beginning to make inferences about distinct components of the inflammatory network in different pathological settings using circulating biomarkers. For example, with a panel of 19 proteins measured in the blood of older adults enrolled in a community-based study, Morrisette-Thomas and colleagues used a principal component analysis to identify two distinct components of immune signaling, both of which were associated with current and future chronic age-related disease: (a) a component characterized by simultaneous coexpression of pro- and antiinflammatory proteins that showed a strong positive correlation with age (top proteins include sTNF-R1, sTNR-R2, IL-6, and TNF-α) and (b) a component characterized by an innate immune response that was not correlated with age (top proteins include MCP, IL-12, IL-8, and MIP) (103). Similar studies have also observed the separation of inflammation into three components (102, 104). With advances in multiplexing, investigators can now measure hundreds of immune proteins and transcripts in various matrices (105, 106). A recent analysis that used bulk RNA sequencing at ten time points in 17 tissues across the mouse lifespan found that genes linked to immune response pathways were among the most differentially expressed with age across organ systems. Two clusters of immune response genes, which included immunoglobulin J chain, β_2_-microglobulin, and complement C1q A chain (C1QA), were upregulated well before other gene clusters during the transition from middle to late life. Changes in the expression of these immune gene networks were proposed to originate from the infiltration of T and B cells into a diverse set of organ systems, most prominently in white adipose tissue (107).

Several studies have characterized changes in multiple biomarkers following an initial immune challenge. An early study that examined the transcriptional expression response to bacterial endotoxin revealed that different functional networks emerge during the response time course (108). For example, some proinflammatory cytokines (TNF-α and IL-1β) and chemokines (CCL2 and CCL10) showed the strongest expression at 2 to 4 hours, whereas the peak expression of antiinflammatory markers occurred at the 4- to 6-hour mark. A downregulation of transcriptional modules linked to mitochondrial bioenergetics and protein synthesis was also noted, highlighting the breadth of biological changes that coincidentally occur with inflammation. The study of temporal dynamics of the inflammatory response to a challenge may uncover a proinflammatory diathesis prior to the development of full-fledged inflammaging.

Single-cell RNA sequencing studies have revealed complex patterns of common and organ-specific immune cell changes with aging in mice and humans (109–111). In particular, leukocytes in advanced age acquire a proinflammatory profile (IL-1β, CD14, TNFRSF12A) and show decreased expression of antiinflammatory markers (e.g., CD9, CD88) (110). Additionally, monocytes and effector memory CD4^+^ T cells become more abundant, whereas plasmacytoid dendritic cells, naive CD4^+^ T cells, and CD8^+^ T cells decline with aging (111–113). An age-related increase in GZMK^+^ CD8^+^ T cells, i.e., a clonal, exhausted-like, granzyme K–expressing T cell, was recently identified as a prominent feature of inflammaging that contributes to increased inflammatory cytokines by promoting the SASP (112).

Developing immune age scores is an essential step to defining inflammaging and its relationship with adverse health outcomes. A recent study of adults used proteins, transcripts, and immune cell counting to construct an immune age (IMM-AGE) score that explained a significant portion of the interindividual cytokine response variability (114). The authors suggested that the biological age of the immune system is a primary determinant of overall inflammatory signaling. The IMM-AGE score was a stronger predictor of mortality than a DNA methylation clock in the same cohort. An inflammatory age score derived from levels of 73 inflammatory plasma and CSF proteins was used to study the relationship between age-related inflammation and dementia risk (115). Proteins involved in the response to cytokine stimulus (e.g., TNFSF14) and chemotaxis (e.g., CCL3, CXCL9) were ranked highest in the inflammatory age score, which accounted for approximately 40% of the variance in chronological age. A higher inflammatory age score was associated with Alzheimer’s disease (AD) dementia diagnosis, AD pathology, and lower cognition, indicating that a well-designed immune age score may have diagnostic and prognostic utility.

Genetics has long been considered one potential determinant of inflammatory aging that operates independent of exogenous factors such as infection and environmental exposures. For example, one large-scale GWAS found a median heritability of 37% for a broad set of immune cell types and immune traits, with some phenotypes showing heritability as high as 96% (116, 117). The heritability for many serum cytokines/chemokines ranges from 50% to 90% in healthy twins between 8 and 82 years of age, with IL-6 and IL-12p40 levels being especially driven by genetics. However, monozygotic twins show age-related divergence of inflammatory biomarker levels, suggesting that the nonheritable influence on inflammation increases with time (118). Like basal cytokine levels, the stimulus-induced cytokine production in response to bacterial, fungal, viral, or a nonmicrobial challenge can also be highly influenced by genetic variation. However, the degree to which genetics contributes to cytokine responses varies considerably according to the inflammatory stimulus, suggesting a genetic influence on trigger-specific responses (119–121). For example, the proportion of variance in TNF-α levels explained by genome-wide SNP data was more than 70% after ex vivo stimulation with fungi and more than 50% after ex vivo stimulation with LPS. While genetic factors explained little of the variation seen in TNF-α levels after stimulation with bacteria, such as *E*. *coli*, a large proportion of variation in IL-6 and IL-22 levels after challenge with bacterial stimuli was determined by genetics (119). Thus, while some aspects of inflammaging are likely driven by genotype, there is also evidence for the role of gene-environment interactions.

The application of multi-omics technology and the emergence of systems immunology has improved our understanding of inflammation and its interaction with the aging process. Many questions remain unsolved; perhaps the most important is how to identify and validate biomarkers for distinct inflammation subtypes (e.g., acute vs. chronic vs. tissue remodeling) and how to specify aspects of inflammation that can be selectively targeted to prevent age-related diseases.

### New proteomic technologies for studying biomarker signatures.

Proteomic technologies, including mass spectrometry–based proteomics, aptamer-based arrays (SomaLogic), and proximity extension assay technology (Olink), are now available for the large-scale quantification and validation of thousands of proteins in human biological fluids or tissues (122–127). Although the advances in aptamer and proximity extension assay technology have significantly expanded access to large-scale proteomics, there remain certain limitations to these approaches, including platform-specific measurement variation, incomplete target validation, a bias toward measurement of secreted proteins, and a limited breadth of protein measurement (128). A full characterization of protein variability also remains a major technical challenge, since the approximately 20,300 human coding genes can produce hundreds of thousands of protein variants through alternative splicing and posttranslational modification (PTM) (129, 130). Though many of these proteins and proteoforms are likely relevant mediators of inflammation, most are not quantified by current large-scale proteomic approaches. Emerging mass spectrometry–based workflows with new computational pipelines as well as recently developed PTM analyses are starting to address this knowledge gap (131, 132). A recent landmark study introduced the Blood Proteoform Atlas, a reference map of proteoforms in 21 cell types in human blood and bone marrow (133). This platform has uncovered that proteoforms have higher cell type specificity than protein-level measurements, laying the groundwork for possible dissection of the different arms of the inflammatory response. By leveraging the specificity of the top-down proteoform with the robustness of bottom-up proteomic approaches (134), a new generation of biomarker signatures will likely emerge that permit accurate profiling and quantification of complex biological phenomena such as aging and inflammaging.

## Inflammation and the aging brain

The brain is an immune-privileged organ with highly regulated innate and adaptive immune processes designed to maintain homeostasis and quickly clear pathogens while limiting exposure to peripheral immune challenges. However, in the context of inflammaging and dysregulation of the neuro-immune axis, the brain becomes increasingly vulnerable. Immune activation, both within and outside of the CNS, has been observed in many age-related neurological diseases. Mechanisms by which age-related inflammation and neuroimmune activation jointly affect brain health may be targets for disease prevention.

### The neuro-immune axis.

Inflammaging can influence homeostatic neurobiological processes and precipitate or perpetuate the development of neuropathology. The blood-brain barrier (BBB) prevents blood molecules from freely entering the CNS and is a key conduit through which inflammatory factors interact with brain targets. The BBB in the brain microvasculature is composed of a continuous endothelial cell layer connected by tight junctions and sheathed by pericytes, basal membranes, and perivascular astrocytes (135). By shielding the brain from pathogens, neurotoxins, and immune molecules, the BBB helps to protect the brain from chemical and biological stressors. BBB permeability becomes less selective with aging because of a loss of tight junctions and a shift from tightly regulated receptor-mediated transcytosis toward nonspecific transcytosis of plasma proteins (136, 137). Age-related inflammatory factors in blood, such as TNF-α, can further increase BBB permeability by suppressing the expression of tight junctions and enhancing brain endothelial cell adhesion, ultimately increasing neuroinflammation (138–140).

**Figure 3:**
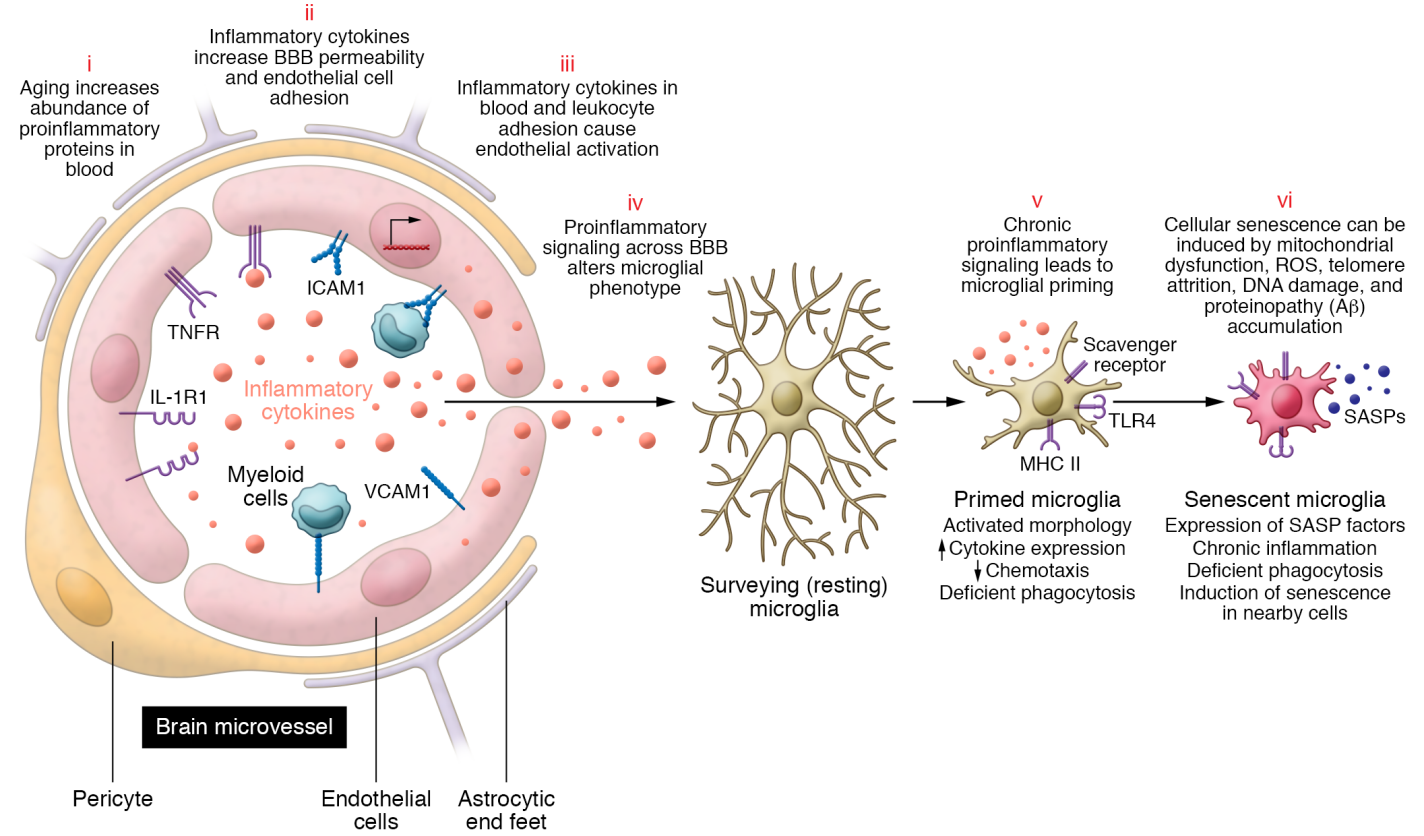
The role of inflammation in neurovascular and brain aging. (i) Aging is associated with tissue-specific increases in proinflammatory protein expression, resulting in greater levels of inflammatory mediators (cytokines and chemokines) in peripheral circulation (blood). (ii) Inflammatory cytokines in blood signal cytokine receptors expressed in brain endothelial cells. This results in increased blood-brain barrier permeability and an upregulation of cellular adhesion molecules such as VCAM1. (iii) Inflammatory cytokines signal cytokine receptors on the luminal side of brain endothelial cells, leading to proinflammatory activation and leukocyte adhesion to vascular adhesion molecules, both of which promote proinflammatory endothelial activation. Together, these processes lead to an increased expression of inflammatory proteins in the brain parenchyma. (iv) Inflammatory proteins in the brain parenchyma can influence microglia and astrocyte phenotypes. For example, initial exposure of microglia to inflammatory mediators causes a transition from a surveilling homeostatic phenotype to an intermediate or activated phenotype. (v) Prolonged exposure to inflammatory signaling is believed to cause long-term microglial priming, characterized by exaggerated cytokine expression, reduced chemotaxis, and deficient phagocytosis. Primed microglia are more prone to aberrant expression in the context of disease. (vi) DNA damage, mitochondrial dysfunction, exposure to proteinaceous aggregates, and other forms of cellular stress can promote senescence of cells within the central nervous system, including brain endothelial cells and microglia. GFAP, glial fibrillary acidic protein; ICAM1, intercellular adhesion molecule 1; IL-1R1, IL-1 receptor type 1.

Circulating TNF-α, IL-1β, and IL-6 bind to receptors in endothelial cells and induce the expression of cellular adhesion proteins (e.g., VCAM1) that further promote inflammation by triggering NF-κB signaling (Figure 3) (136, 140, 141). This process facilitates tethering of circulating myeloid cells to the brain endothelia, augmenting the intravascular inflammatory state and promoting the activation of microglia, the brain’s innate immune cell (140). In parallel, the entry of inflammatory cytokines and chemokines occurs through active transport or nonspecific caveolar transcytosis (137, 142). Inflammatory proteins may enter the brain through other channels as well, including via the choroid plexus and circumventricular organs (143, 144).

### Peripheral inflammation, brain aging, and disease.

Many inflammatory mediators known to increase with aging (IL-1β, IL-18, sTNF-R1) are upregulated to an even greater extent in neurodegenerative conditions such as AD and Parkinson’s disease (PD) (100, 145–147). Studies have demonstrated that a proinflammatory state (148–150), inflammatory disease (e.g., rheumatoid arthritis) (151), and immune challenges (e.g., acute infection) (152, 153) all increase the risk for dementia over multiple decades. Genetic studies further support the role of immunity and inflammation in conditions such as AD, PD, and amyotrophic lateral sclerosis (ALS). For example, many polymorphisms associated with late-onset sporadic AD are in or near genes important for immunity, such as *TREM2*, *CD33*, *CR1*, *GRN*, and *IL1RAP*, and/or are expressed in brain immune cells including microglia (154). Counterintuitively, higher levels of IFN-γ and IL-12 (Th1 cytokines) were recently associated with slower cognitive decline in older adults, suggesting that the contribution of inflammatory signaling to neurodegenerative disease is complex and likely dependent on the inflammatory mediator in question (153).

### Neuroinflammation, aging, and disease.

Neuroinflammation, a CNS-specific process characterized by proinflammatory cellular and molecular changes to microglia, astrocytes, and the BBB, is considered a core feature of aging and age-related neurological conditions, most notably AD, PD, and ALS (155). Age itself is associated with phenotypic changes to microglia, including deramified morphology, increased cytokine and chemokine expression, and an upregulation of MHC II and TLRs (156, 157). These changes, often referred to as microglial priming or sensitization, result in an amplified and prolonged glial inflammatory response after both central and peripheral immune challenges (158, 159). Astrocytes also demonstrate similar age-related changes, including elevated glial fibrillary acidic protein (GFAP) expression and hypertrophic morphology (160), both of which suggest a transition toward a proinflammatory state.

Aged glial cells respond less efficiently to antigens, including amyloid-β (Aβ) and α-synuclein, and have reduced phagocytic and antiinflammatory capacity (161–163). Delayed resolution of inflammation makes the brain particularly vulnerable to aberrant microglial responses to acute stressors, such as infection or tissue injury (164, 165). Microglia and astrocytes can also be primed and activated by age-related neuropathological processes, such as cerebrovascular lesions and protein aggregates (165, 166). Cellular senescence is yet another factor that can augment glial function and accelerate brain aging. Microglia, astrocytes, brain endothelial cells, and other CNS cell types show evidence of senescence and increased expression of senescence factors (167, 168). The expression of senescent factors, SASPs in particular, creates an inflammatory milieu within the brain believed to modulate neuroimmune processes while also compromising the integrity of the BBB (169, 170). The aggregation of misfolded proteins, including Aβ plaques and tau neurofibrillary tangles, may also promote neuronal and glial senescent phenotypes (171), as do other common facets of neurodegeneration, such as mitochondrial dysfunction and abnormal proteostasis (22, 172, 173). Although less direct, cellular senescence within the periphery and its associated SASP may also prime microglia and affect neurodegenerative processes through the immune-brain axis. Though each of these biological processes has been linked to neurodegeneration and cognitive decline, a causal role for cellular senescence in neurodegenerative disease has yet to be established. Still, many contend that removal of senescent cells within the CNS may be neuroprotective in older adults, particularly in the context of AD and PD (23, 174).

The extent to which neuroinflammation is either protective or pathogenic in the context of age-related changes is unknown and likely dependent on disease etiology and disease stage. For example, in AD and frontotemporal dementia, neuroinflammation and microglia activation potentiate the spreading of pathological tau neurofibrillary tangles (83, 175). In AD, this relationship may be mediated by NLRP3 inflammasome activation in microglia, a process that activates tau kinases (e.g., GSK-3β and CaMKII-α) and results in tau hyperphosphorylation (83). However, microglial activation may also be protective in these neurodegenerative conditions, for example by enhancing microglia phagocytosis of amyloid plaques in the context of AD (176–178). Much remains to be learned about how inflammaging and neuroinflammation interact to influence brain health and neurodegenerative disease.

## Conclusions

Understanding the causes and consequences of inflammaging is one of the most active areas of research in aging and chronic diseases and one with powerful potential for translation. Work on biomarkers searching for signatures that discriminate between different aspects and triggers of inflammation is currently ongoing. Despite tremendous progress, the robustness and generalizability of developed signatures are not ready for clinical applications (179). Refining and validating these signatures in both animal models and humans and connecting them with specific biological mechanisms, age-related chronic diseases, and health outcomes remains a central research goal. An important question is whether signatures of circulating biomarkers will identify inflammatory processes that occur in different tissues and from different pathogenetic mechanisms. Progress in this field will require technological improvements in the measurement and analysis of multi-omics and rapid cycles of translation from model organisms to humans and vice versa to identify new promising therapeutic targets.

## Version 1 07/15/2022

Electronic publication
